# Rural and Urban Differences in Prostate Cancer Recurrence

**DOI:** 10.1001/jamanetworkopen.2025.26912

**Published:** 2025-08-08

**Authors:** Julia B. Balmaceda, Aaron J. Katz, Ying Cao, Katelyn Kane, Matthew W. Chen, Deborah Usinger, Xinglei Shen

**Affiliations:** 1 Department of Internal Medicine, University of Kansas Medical Center, Kansas City; 2 Department of Radiation Oncology, University of Kansas Medical Center, Kansas City; 3 Department of Population Health, University of Kansas Medical Center, Kansas City; 4 Department of Urology, University of North Carolina at Chapel Hill, Chapel Hill

## Abstract

**Importance:**

Rural patients face unique barriers in obtaining high-quality cancer care. Research is lacking in determining whether these disparities translate to negative clinical outcomes.

**Objective:**

To determine if there are differences in the rate of biochemical recurrence in prostate cancer between rural and urban men.

**Design, Setting, and Participants:**

This cohort study used patient data that was abstracted from the North Carolina Prostate Cancer Comparative Effectiveness and Survivorship Study, a population-based cohort that prospectively followed patients with newly diagnosed prostate cancer from the Rapid Case Ascertainment system of the North Carolina Central Cancer Registry from January 1, 2011, to June 30, 2013. Patients were identified who had received either surgery or radiation within 1 year of diagnosis. Data were analyzed from January 2011 to December 2022.

**Exposure:**

Patients were categorized as rural or urban using the Rural-Urban Continuum Code.

**Main Outcome and Measures:**

Recurrence was determined by prostate-specific antigen testing. Demographic differences between rural and urban patients were assessed using 2-sample independent *t* test and χ^2^ test. Prostate cancer recurrence was analyzed using Cox proportional hazard models.

**Results:**

A total of 778 patients were included with a median (IQR) follow-up of 4.6 (2.0 to 6.9) years and a mean (SD) age of 63 (7.4) years. Additionally, 213 were Black men (27.4%), 565 were White men (72.6%), 350 were Medicare insured (45.1%), 324 had an income ranging from $40 000 to $90 000 (43.1%), 370 were a National Comprehensive Cancer Network (NCCN) intermediate risk group (47.6%), 449 were treated with radical prostatectomy (57.7%), and 690 were in good to excellent health (88.7%) with 191 living in a rural setting (24.6%). On univariable analysis, rural residence (hazard ratio [HR], 2.19 [95% CI, 1.38 to 3.46]; *P* < .001), NCCN risk group (HR, 4.13 [95% CI, 2.25 to 7.57]; *P* < .001), and having had fewer than 12 biopsies (HR, 1.70 [95% CI, 1.08 to 2.67]; *P* = .02) were significantly associated with biochemical recurrence. On multivariable analysis adjusted for location of residence, marital status, overall health, number of cores biopsied, NCCN risk group, and treatment type, rural residence was significantly associated with recurrence (HR, 1.74 [95% CI, 1.07 to 2.82]; *P* = .03), while radiation therapy was inversely associated with recurrence (HR, 0.51 [95% CI, 0.31 to 0.85]; *P* = .01).

**Conclusions and Relevance:**

In this cohort study of patients with newly diagnosed prostate cancer, rural patients with prostate cancer had higher rates of biochemical recurrence. The etiology of this disparity is unclear but is likely multifactorial. Factors that may play a role include socioeconomic status, delay and disruptions in care, and access to multidisciplinary cancer care.

## Key Points

**Question:**

Are there differences in the rate of prostate cancer biochemical recurrence between rural and urban patients?

**Findings:**

In this cohort study that included 778 patients with prostate cancer, rural patients had significantly higher rates of recurrence at 16.8% compared with urban patients at 8.5%.

**Meaning:**

These findings suggest that there is need for more research to evaluate the causes of this observation and possible interventions to combat this disparity.

## Teaser

This cohort study examines differences in the rate of biochemical recurrence of prostate cancer among rural vs urban men.

## Introduction

Prostate cancer is the most common cancer among men in the US. Like many diseases, health disparities can be seen in prostate cancer. It is well documented that health disparities are a summation of biases in our health care system, genetic and epigenetic factors, and accessibility to care.^1,2,3^ Patients living in a rural setting often come from lower socioeconomic backgrounds, travel longer distances to receive cancer treatment, and face occupational exposures that may contribute to poorer health outcomes.^4,5^ Patients from rural settings are also less likely to undergo cancer screening^6^ and are offered treatment options at different rates than patients in urban settings, which may negatively impact the quality of cancer care and related outcomes, including cancer recurrence.^7,8^

Prior work has revealed differences in prostate cancer screening, access to treatment, and treatment choice between rural and urban populations.^9^ There are lower concentrations of clinicians in rural regions. Greater distance to treatment facility is associated with decreased likelihood of curative treatment or treatment that deviates from national guidelines.^10,11^ Most prior studies were performed using population-based databases, which were able to compare differences in trends in survival but do not have the ability to detect disparity in recurrence, as this end point is not captured in cancer registry data from which databases are derived.^12,13^ No studies have examined whether the differences in treatment, screening, and access have a significant clinical implication on recurrence of prostate cancer.

Understanding disparities in recurrence is important. The earliest way to detect recurrence is through the rise of a biomarker, prostate-specific antigen (PSA) after treatment, a condition called biochemical recurrence. The incidence of biochemical recurrence ranges from 14% to 46%.^14,15,16^ Risk factors for recurrence include high pathologic staging, high Gleason score, extraprostatic extension, positive surgical margins, and elevated PSA doubling time.^17^ Some studies have associated African American race, geographic location, and public health insurance with recurrence.^16,18,19^ Biochemical recurrence may ultimately lead to metastasis and death from prostate cancer. Importantly, salvage therapy for recurrent prostate cancer has major negative effects on patient quality of life ranging from sexual dysfunction to urinary and bowel incontinence.^20,21^ Biochemical recurrence also has direct and indirect economic implications for patients due to resource-intensive treatments, drug cost, and the health care utilization costs associated with managing long-term complications of salvage therapy.^22,23^ Thus, identifying differences in prostate cancer recurrence between men in rural and urban settings may lead to and support efforts to improve the quality of prostate cancer care among rural patients and address disparities in quality of life and health outcomes.

We hypothesize that rurality is associated with higher recurrence after standard treatment for prostate cancer. We used the North Carolina Prostate Cancer Comparative Effectiveness and Survivorship Study (NC ProCESS) to evaluate biochemical recurrence by rural or urban status.

## Methods

### Patient Population

NC ProCESS is a population-based cohort of men enrolled soon after diagnosis and followed prospectively. In collaboration with the North Carolina Central Cancer Registry, from January 1, 2011, to June 30, 2013, newly diagnosed prostate cancer patients from all counties across North Carolina were contacted to participate. Tumor registrars at local hospitals provided pathologic and diagnostic information. Patients were enrolled prior to treatment and followed prospectively to collect medical records and patient-reported outcomes. For each patient, records from their primary care physician, urologist, and radiation oncologist, if consulted, were requested. Details of study design and the cohort were described previously.^24^ Baseline data were collected prior to starting treatment. All study participants provided written informed consent and data were deidentified. This cohort study was approved by the University of North Carolina and University of Kansas Institutional Review Boards. The authors followed the Strengthening the Reporting of Observational Studies in Epidemiology (STROBE) reporting guideline.^25^

The goal of the current analysis is to determine if there are urban vs rural differences in prostate cancer recurrence after definitive therapy. Therefore, patients analyzed are those who received definitive treatment with either radical prostatectomy or radiation therapy (external beam radiation or brachytherapy) within 1 year of diagnosis. Patients treated with Cyberknife were excluded. Only patients identifying as Black or White were included in this analysis. The initial study population consisted of 1413 patients enrolled in NC ProCESS; of these, 778 met the inclusion criteria for this analysis.

### Data Collection

Patient demographic information was collected by self-report at study enrollment. Sociodemographic data included race, income, education level, employment status, insurance status, and marital status. Subjective overall health status was self-reported. Patients were classified as rural using the US Department of Agriculture 2013 Rural-Urban Continuum Code (RUCC) for the county of primary residence. Consistent with prior work using the RUCC to identify rural patients with cancer and the National Cancer Institute’s definition, participants who lived in RUCC 1 to 3 areas were categorized as urban and those who lived in RUCC 4 to 9 areas as rural.^26,27^ Prospective follow-up included data collection from patient report, medical records, and data from the North Carolina Central Cancer Registry. Both medical records and cancer registry data were used for details on treatment and prostate cancer diagnosis (PSA, clinical stage, Gleason score, National Comprehensive Cancer Network [NCCN] risk group, and age at diagnosis). Medical records were abstracted for development of recurrence after primary treatment. Recurrence was defined per published guidelines for radical prostatectomy (PSA greater than 0.2 ng/mL)^28^ and for radiation therapy (2 ng/mL above the postradiation therapy nadir).^29^

### Statistical Analysis

Data were included from January 2011 to December 2022. Descriptive statistics including 2-sample independent *t* test and χ^2^ test were used to assess demographic differences between rural and urban patients. Univariable analysis and multivariable analysis using Cox proportional hazards models assessed the association between urban vs rural residence with development of prostate cancer recurrence. The multivariable model was formed by first performing a univariable analysis, followed by a stepwise, forward and backward variable selection. This compares the AIC (Akaike Information Criterion) scores among the models as criteria to choose best combinations of variables for our final multivariable model. The final set of variables, or the final models, given the data and the AIC criterion as well as stepwise (backward, forward or both) procedure, provide the best trade-off between model fit and complexity. The Cox proportional hazards were tested using the Supremum Test with 1000 simulations to test the proportional hazards assumption with *P* > .05 as well as visually assessing the assumptions via Schoenfeld Residual plot.

To assess the quality of data, address potential ascertainment bias and potential biases from missing data, we performed analyses of mean follow-up time and rate of PSA testing between rural and urban groups. Short follow-up was defined as follow-up less than 2 years. All statistical analyses were performed using SAS version 9.4 (SAS Institute Inc), and a *P* < .05 was deemed statistically significant.

## Results

A total of 778 patients were included with a median (IQR) follow-up of 4.6 (2.0 to 6.9) years and a mean (SD) age of 63 (7.4) years. Additionally, 213 were Black men (27.4%), 565 were White men (72.6%), 350 were Medicare insured (45.1%), 324 had an income ranging from $40 000 to $90 000 (43.1%), 370 were an NCCN intermediate risk group (47.6%), and 690 were in good to excellent health (88.7%) with 191 living in a rural setting (24.6%) (Table 1). Overall, 449 patients underwent radical proctectomy (57.7%) and the remaining 329 patients were treated with radiation therapy (42.3%). There were no statistically significant differences in sociodemographic factors between rural and urban patients. NCCN risk groups were distributed similarly across rural and urban patients, but rural patients were more likely to have fewer than 12 cores assessed at biopsy than urban patients (97 of 191 [51.1%] vs 215 of 587 [36.6%]; *P* = .004). Overall, urban and rural patients had mean (SD) follow-up times of 4.6 (2.8) years and 4.3 (2.7) years, respectively (*P* = .17). There was no significant difference in the proportion of patients with shorter follow-up (ie, less than 2 years) between rural and urban patients (29 of 182 [26.9%] vs 133 of 562 [23.7%]; *P* = .37).

**Table 1.** Baseline Characteristics of Patient Cohort

Characteristic	Total (n = 778)	Urban (n = 587)	Rural (n = 191)	*P* value
Age at diagnosis, mean (SD), y	62.8 (7.4)	62.6 (7.4)	63.4 (7.4)	.22
Race				
Black	213 (27.4)	156 (26.6)	57 (29.8)	.38
White	565 (72.6)	431 (73.4)	134 (70.2)	
Education				
High school or less	248 (31.9)	179 (30.5)	69 (36.1)	.15
Any college or more	530 (68.1)	408 (69.5)	122 (63.9)	
Employment				
Yes	362 (46.5)	282 (48.0)	80 (41.9)	.14
No	416 (46.5)	305 (52.0)	111 (58.1)	
Household income				
<$40 000	256 (34.1)	197 (34.8)	59 (31.9)	.053
$40 000 to <$90 000	324 (43.1)	231 (40.8)	93 (50.3)	
≥$90 000	171 (22.8)	138 (24.4)	33 (17.8)	
Insurance				
Medicare	350 (45.1)	253 (43.1)	97 (51.3)	.14
Private insurance	319 (41.1)	250 (42.6)	69 (36.5)	
Uninsured or other coverage	107 (13.8)	84 (14.3)	23 (12.2)	
Marital status				
Married	622 (79.9)	464 (79.0)	158 (82.7)	.27
Not married	156 (20.1)	123 (21.0)	33 (17.3)	
Overall health				
Fair or poor	88 (11.3)	67 (11.4)	21 (11.0)	.72
Good	223 (28.7)	174 (29.6)	49 (25.7)	
Very good	304 (39.1)	226 (38.5)	78 (40.8)	
Excellent	163 (21.0)	120 (20.4)	43 (22.5)	
No. of cores biopsied				
<12	312 (40.2)	215 (36.6)	97 (51.1)	.004^a^
≥12	465 (59.8)	372 (63.4)	93 (48.9)	
Treatment				
Radiation therapy	329 (42.3)	245 (41.7)	84 (44.0)	.59
Radical prostatectomy	449 (57.7)	342 (58.3)	107 (56.0)	
NCCN risk group				
Low	289 (37.1)	219 (37.3)	70 (36.6)	.39
Intermediate	370 (47.6)	284 (48.4)	86 (45.0)	
High	119 (15.3)	84 (14.3)	35 (18.3)	

^a^ Difference was statistical signifcant at *P* < .05.

Overall, 82 patients (10.5%) experienced biochemical recurrence with a median (IQR) follow-up of 4.6 (2.0 to 6.9) years after treatment. On univariable analysis (Table 2), rural patients had significantly higher rates of recurrence compared with urban patients (16.8% vs 8.5%; hazard ratio [HR], 2.19 [95% CI, 1.38 to 3.46]; *P* < .001). Other factors associated with recurrence included NCCN high risk group (HR, 4.13 [95% CI, 2.25-7.57]; *P* < .001) and having fewer than 12 cores biopsied (HR, 1.70 [95% CI, 1.08-2.67]; *P* = .02). Interestingly, radiation therapy was associated with a lower risk of recurrence compared with radical prostatectomy (HR, 0.58 [95% CI, 0.36 to 0.96]; *P* = .03).

**Table 2.** Univariable Analysis of Factors Associated With Prostate Cancer Recurrence

Covariate	Participants, No. (%)	Hazard ratio (95% CI)	*P* value
Location of residence			
Rural	182 (24.5)	2.19 (1.38-3.46)	<.001^a^
Urban	562 (75.5)	1 [Reference]	
Age at diagnosis			
Per year	744 (100)	1.00 (0.97-1.03)	.97
Race			
Black	198 (26.6)	1.08 (0.65-1.80)	.76
White	546 (73.4)	1 [Reference]	
Education			
High school or less	234 (31.5)	0.70 (0.41-1.21)	.20
Any college or above	510 (68.5)	1 [Reference]	
Employment			
No	398 (53.5)	1.08 (0.65-1.80)	.76
Yes	346 (46.5)	1 [Reference]	
Household income			
<$40 000	237 (33.0)	0.69 (0.37-1.28)	.49
$40 000 to <$90 000	314 (43.7)	0.86 (0.50-1.48)	
≥$90 000	168 (23.4)	1 [Reference]	
Insurance			
Private insurance	301 (40.5)	0.98 (0.60-1.62)	.47
Uninsured or other coverage	104 (14.0)	1.42 (0.76-2.67)	
Medicare	338 (45.5)	1 [Reference]	
Marital status			
Not married	142 (19.1)	0.54 (0.25-1.17)	.12
Married	602 (80.9)	1 [Reference]	
Overall health			
Fair or Poor	82 (11.0)	0.35 (0.12-1.00)	.16
Good	209 (28.1)	0.62 (0.34-1.15)	
Very good	294 (39.5)	0.72 (0.42-1.22)	
Excellent	159 (21.4)	1 [Reference]	
Number of cores biopsied			
<12	296 (39.8)	1.70 (1.08-2.67)	.02^a^
≥12	447 (60.2)	1 [Reference]	
NCCN risk group			
Low	277 (37.2)	1 [Reference]	<.001^a^
Intermediate	358 (48.1)	1.60 (0.90-2.84)	
High	109 (14.7)	4.13 (2.25-7.57)	
Treatment type			
Radiation therapy	302 (40.6)	0.58 (0.36-0.96)	.03^a^
Radical prostatectomy	442 (59.4)	1 [Reference]	

^a^ Difference was statistically significant at *P* < .05.

The mean time to recurrence for both rural and urban patients was 3.2 years (*P* = .94). Prior to the event of recurrence, PSA testing was similar between rural and urban patients with a mean of 2.8 and 3.0 tests per year, respectively (*P* = .60). Among those without recurrence, mean (SD) follow-up time was 4.5 (2.8) years for rural patients and 4.7 (2.8) years for urban patients.

On multivariable analysis (Table 3), rural residence (HR, 1.74 [95% CI, 1.07 to 2.82]; *P* = .03), NCCN risk group (*P* < .001), and treatment (radiation therapy vs radical prostatectomy: HR, 0.51 [95% CI, 0.31 to 0.85]; *P* = .01) remained statistically significantly associated with prostate cancer recurrence.

**Table 3.** Multivariable Analysis of Factors Associated With Prostate Cancer Recurrence

Covariate	Hazard ratio (95% CI)	*P* value
Location of residence		
Rural	1.74 (1.07-2.82)	.03
Urban	1 [Reference]	
Marital status		
Not married	0.53 (0.24-1.19)	.12
Married	1 [Reference]	
Overall health		
Fair or poor	0.38 (0.13-1.12)	.19
Good	0.59 (0.32-1.11)	
Very good	0.69 (0.40-1.18)	
Excellent	1 [Reference]	
No. of cores biopsied		
<12	1.60 (1.00-2.57)	.050
≥12	1 [Reference]	
NCCN risk group		
Intermediate	1.68 (0.94-2.98)	<.001^a^
High	3.99 (2.15-7.41)	
Low	1 [Reference]	
Treatment		
Radiation therapy	0.51 (0.31-0.85)	.01^a^
Radical prostatectomy	1 [Reference]	

Abbreviation: NCCN, National Comprehensive Cancer Network.

^a^ Difference was statistical signifcant at *P* < .05.

In assessing factors associated with prostate cancer recurrence among the subset of rural patients, only employment status at baseline was associated with greater risk (unemployed vs employed: HR, 2.32 [95% CI, 1.09 to 5.48]; *P* = .04) (Table 4). The effect estimate for NCCN risk group was similar to that observed for the overall study sample but was not statistically significant.

**Table 4.** Univariable Analysis of Factors Associated With Prostate Cancer Recurrence in Rural Patients

Covariate	Participants, No. (%)	Hazard ratio (95% CI)	*P* value
Age at diagnosis			
Per year	182 (100)	1.00 (0.96-1.05)	.88
Race			
Black	53 (29.1)	1.27 (0.58-2.62)	.53
White	129 (70.9)	1 [Reference]	
Education			
High school or less	64 (35.2)	1.52 (0.72-3.09)	.26
Any college and above	118 (64.8)	1 [Reference]	
Employment			
No	106 (58.2)	2.32 (1.09-5.48)	.042
Yes	76 (41.8)	1 [Reference]	
Household income			
<$40 000	54 (30.7)	1.56 (0.51-5.41)	.63
$40 000 to <$90 000	90 (51.1)	1.69 (0.65-5.45)	
≥$90 000	32 (18.2)	1 [Reference]	
Insurance			
Medicare	93 (51.4)	1 [Reference]	.65
Private insurance	65 (35.9)	0.75 (0.33-1.70)	
Uninsured or other coverage	23 (12.7)	1.22 (0.44-3.37)	
Marital status			
Not married	30 (16.5)	0.12 (0-0.88)	.15
Married	152 (83.5)	1 [Reference]	
Overall health			
Fair or poor	19 (10.4)	0.81 (0.15-2.96)	.72
Good	46 (25.3)	0.66 (0.21-1.91)	
Very good	75 (41.2)	1.18 (0.52-2.89)	
Excellent	42 (23.1)	1 [Reference]	
No. of cores biopsied			
<12	91 (50.3)	0.82 (0.39-1.70)	.61
≥12	90 (49.7)	1 [Reference]	
NCCN risk group			
Intermediate	83 (45.6)	1.22 (0.53-2.94)	.13
High	31 (17.0)	2.42 (0.97-6.05)	
Low	68 (37.4)	1 [Reference]	
Treatment type			
Radiation therapy	76 (41.8)	0.61 (0.28-1.26)	.20
Radical prostatectomy	106 (58.2)	1 [Reference]	

^a^ Difference was statistical signifcant at *P* < .05.

## Discussion

This study observed that patients from a rural setting were more likely to have biochemical recurrence of their prostate cancer compared with their urban counterparts. Based on our analysis of follow-up time and rate of PSA testing, we do not believe that these factors biased the identification or rurality as a risk factor for recurrence. Our work suggests that geographic disparity contributed to worse prostate cancer control in rural patients. To our knowledge, there have been no other population-based studies that have found rurality as an independent associated factor of prostate cancer recurrence after treatment.

Prior studies have found disparity in mortality between rural and urban patients with certain cancers, such as lung, breast and prostate cancer.^30,31,32,33^ Rural patients are often diagnosed with more advanced disease with this trend being most pronounced for cancers with standardized screening for early detection such cervical cancer and lung cancer.^34^ These studies have been limited in assessment of cancer outcomes to survival end points only as they have generally relied on cancer registry data which do not typically document recurrence. One report found that rural residence in Manitoba had 3.47 higher risk of local recurrence in colorectal cancer.^35^ A retrospective study found that nonadherence to adjuvant therapy was associated with higher rates of recurrence in rural women with breast cancer compared with their urban counterparts.^36^ Additionally, Xuan et al^36^ found that rural women with breast cancer were more likely to be diagnosed with more extensive disease. Currently, the data on the association between rurality and cancer recurrence remains limited. NC ProCESS was able to overcome this limitation by obtaining individual follow-up data to allow for determination of recurrence.

The reasons for worse prostate cancer control in rural patients may be associated with disparity in access and quality.^9^ A similar study performed by Maganty et al^37^ found that nonurban patients were significantly less likely to receive any treatment regardless of their disease risk stratification. Maganty et al^37^ also observed that urban patients were more likely to have private insurance and were more likely to elect surgery whereas nonurban patients more likely to have Medicaid and elect no treatment or observation. Our previous work analyzing this cohort also demonstrated significantly reduced access to multidisciplinary consultations for their cancer care.^9^

One study found that rural patients who experienced delay or disruptions in their radiation treatment were twice as likely to die from their cancer compared with non-rural patients with similar delay or disruptions; the cancer sites in this study included breast, prostate, lung, CNS, head and neck as well as several others.^38^ Another study found that rural patients who experienced delays in their radiation therapy were more likely to be noncompliant with their treatment schedule.^38^ Overall, rural patients were more likely to have nonadherence to their radiation treatment which was associated with poorer survival. In addition to access, the quality of care is worse in rural patients. These patients are more likely to have had inadequate number of cores sampled at biopsy.^9^ Patients with prostate cancer treated at low volume centers also have been shown to have worse cancer outcomes, meaning that although a patient may have access to care, the quality may differ depending on the volume of patients a cancer center treats.^39^

The identification of unemployment as a significant factor underscores the importance of consideration for socioeconomic differences when strategizing efforts to improve cancer outcomes in this patient population.^40^ Although our analysis did not find insurance status as a risk factor for recurrence in the rural group, employment and access to insurance are linked in the US health care system. The cost associated with cancer care can contribute to nonadherence and inadequate follow-up leading to recurrence and overall worse cancer outcomes in this population. North Carolina adopted Medicaid Expansion in 2023. This program will improve access to insurance for poorer and underemployed residents who are overrepresented in our rural cohort.^26,41^ Removing barriers that contribute to nonadherence and access to care may benefit this population.

Future work should look to increase access to quality cancer care for rural patients. Possible solutions should include expanding telehealth options and working to create and expand patient transportation services for those living in locations with limited public transportation. Coordinating care with primary care physicians should be at the core of monitoring rural patients with prostate cancer. These physicians tend to have long-term relationships with these patients and are usually easier for patients to follow-up with compared with their oncologist.^42,43^ Institutions should also consider expanding educational opportunities for trainees in rural areas. This exposes medical students and residents to the unique care infrastructure that rural residents must operate in. Lastly, there is a clear disparity in rural patient accrual in clinical trials^44^; thus, institutions should target enrollment in these areas to further understand the driving factors of this disparity in prostate cancer recurrence.

### Limitations

This study has several limitations. These include the use of self-reported patient demographic data and possibly limited generalizability due to collecting data in only 1 state. The database used medical records provided by hospitals, primary care physicians and specialists which may not be complete; thus, some treatment and follow-up data may be missing. Our analysis of risk factors for recurrence among rural patients was limited by sample size. We examined rurality using a dichotomous variable based on patient zip codes, however, we were unable to analyze distance to treatment center due to insufficient data. Lastly, length of follow-up was variable which may lead to an underestimation of biochemical recurrence. However, we do not believe any potential underestimation of biochemical recurrence differed by patient or clinical characteristics.

## Conclusions

This study uniquely identifies rurality as a risk factor for biochemical recurrence in prostate cancer. We used a large patient cohort with long-term follow-up data that assessed multiple potential risk factors. Our findings suggest that urgent efforts are necessary to address this disparity, including further investigation into modifiable causes of poorer cancer outcomes such as access to insurance among rural patients and funding for programs designed to improve rural health and access.

## Notes

Supplement.
Data Sharing Statement
