# Supplementary material for: Rural and Urban Differences in Prostate Cancer Recurrence
Source: JAMA Netw Open. 2025 Aug 8;8(8):e2526912. doi: 10.1001/jamanetworkopen.2025.26912 (PMC12334958; doi:10.1001/jamanetworkopen.2025.26912)
Supplement: Supplement. — Data Sharing Statement [file jamanetwopen-e2526912-s001.pdf]

Data Sharing Statement

Balmaceda. Rural and Urban Differences in Prostate Cancer Recurrence. *JAMA Netw Open*. Published August 07, 2025. doi:10.1001/jamanetworkopen.2025.26912

Data
Data available: No
